# Cuticular drusen presenting with central serous chorioretinopathy in both eyes: A case report

**DOI:** 10.1097/MD.0000000000032032

**Published:** 2022-12-02

**Authors:** Shuhei Hosoda, Yoichi Sakurada, Taiyo Shijo, Kenji Kashiwagi

**Affiliations:** a Department of Ophthalmology, Faculty of Medicine, University of Yamanashi, Yamanashi, Japan.

**Keywords:** central serous chorioretinopathy, cuticular drusen, photodynamic therapy

## Abstract

Drusen are precursor lesions to advanced age-related macular degeneration. Although cuticular drusen are located between the retinal pigment epithelium and Bruch’s membrane, as are conventional drusen, they possess unique characteristics that are distinct from those of conventional drusen on clinical presentations. Central serous chorioretinopathy (CSC) is a rare complication in eyes with cuticular drusen.

**Patient concern:**

A 58-years-old man was referred to our institute for the treatment of persistent subretinal fluid (SRF) in both eyes. Spectral-domain optical coherence tomography revealed focal SRF that did not involve the central macula of the right eye and SRF in the central macula of the left eye. Fluorescein angiography exhibited focal leakage corresponding to SRF and hyperfluorescence resembling a “stars in the sky” appearance in both eyes. On initial presentation, the best-corrected visual acuity values were 1.2 and 0.9 in the right and left eye decimal formats, respectively.

**Diagnosis:**

Cuticular drusen presenting with CSC in both eyes.

**Interventions:**

No treatment was administered for CSC in the right eye, whereas photodynamic therapy was administered for CSC in the left eye.

**Outcomes:**

At the 6-month visit, extrafoveal SRF persisted in the right eye and resolved in the left eye. Best-corrected visual acuity improved from 0.9 to 1.2 in the decimal format in the left eye.

**Lessons:**

Although cuticular drusen presenting with CSC are rare, physicians should be aware of the possibility of CSC development in eyes with cuticular drusen.

## 1. Introduction

Drusen are extracellular deposits between the retinal pigment epithelium and Bruch’s membrane and are considered precursor lesions to age-related macular degeneration (AMD), although small drusen are age-related changes. Therefore, they do not increase the risk of AMD, including macular neovascularization (MNV) and geographic atrophy (GA).^[1,2]^

Among several drusen types, cuticular drusen possess distinct characteristics from conventional drusen.^[3,4]^ Gass first described cuticular drusen as small, round, yellow lesions predominantly distributed in early adulthood in the macula and/or peripheral retina. Gass^[5]^ initially reported that they comprised focal nodular thickening of the retinal pigment epithelium basal lamina and named them “basal laminar drusen.” Later, a histopathologic correlation study demonstrated that basal laminar drusen are located between the retinal pigment epithelium (RPE)-basal lamina and Bruch’s membrane, as are conventional drusen.^[6]^ Therefore, the term “basal lamina drusen” is no longer in use. The term “cuticular” derives from the “cuticular” layer between the basal lamina of the RPE and inner collagenous layer of Bruch’s membrane.

Although no epidemiological studies have investigated the prevalence of cuticular drusen using multimodal imaging, they predominantly occur in Caucasians, and reports on cuticular drusen in Asians are limited.^[7–9]^ Apart from the characteristics mentioned above, cuticular drusen possess unique characteristics, including early onset and an increased risk of acquired vitelliform lesions in addition to MNV and GA.^[10,11]^

However, no reports have documented macular complications in eyes with cuticular drusen other than the 3 abovementioned entities. In this report, we describe a case of cuticular drusen with central serous chorioretinopathy (CSC) in both eyes.

## 2. Case report

**Figure 1.**
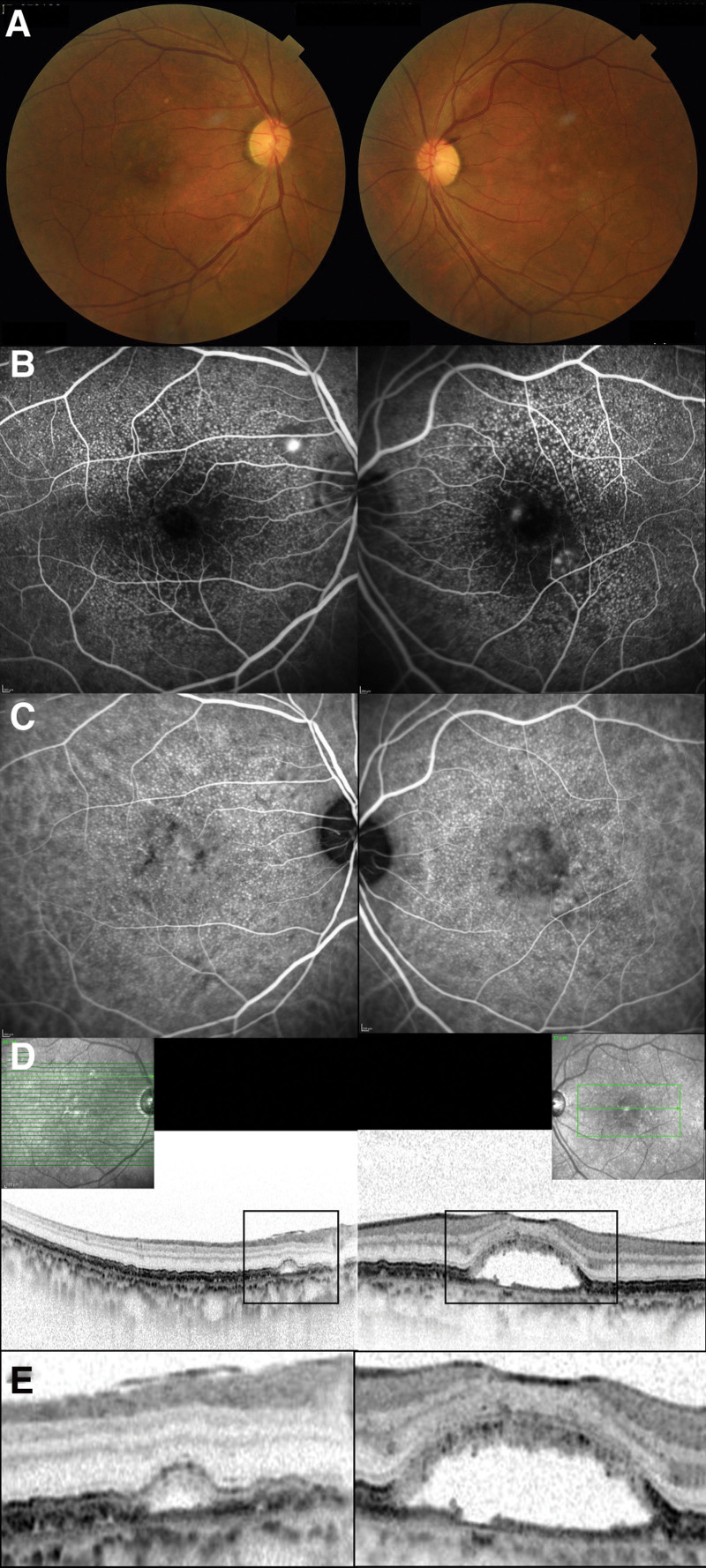
Multimodal imaging on initial presentation. (A) Color fundus photography revealed retinal pigment abnormalities in both eyes. (B) Mid-phase fluorescein angiography (FA) revealed extensive hyperfluorescence resembling a “stars in the sky” appearance in both eyes and focal leakage in the peripapillary and central macula regions of the right and left eyes, respectively. (C) Mid-phase indocyanine green angiography exhibited widespread hyperfluorescence in both eyes. (D) Spectral-domain optical coherence tomography (SD-OCT) covering the macular region demonstrated the presence of subretinal fluid in the peripapillary and central macular regions corresponding to the focal leakage on FA in the right and left eyes, respectively. The central retinal thicknesses were 197 and 359 μm in the right and left eyes, respectively. The subfoveal choroidal thicknesses were 228 and 237 μm in the right and left eyes, respectively. (E) Magnified SD-OCT revealed persistent subretinal fluid with a retinal pigment epithelium (RPE) defect in the left eye; however, RPE defects were not observed in the right eye.

A 58-year-old male patient initially presented with visual deterioration in his left eye. The best-corrected visual acuity (BCVA) values for the patient’s right and left eyes were 1.2 and 0.9 in the decimal format, respectively. On initial presentation, a comprehensive examination was performed, including color fundus photography, fluorescein and indocyanine green angiography (FA/ICGA), spectral-domain optical coherence tomography (SD-OCT), and near-infrared reflectance using Spectralis^®^ (Fig. 1). Color fundus photography revealed RPE abnormalities in both eyes. On FA, hyperfluorescence resembling a “stars in the sky” appearance was observed around the macular area. Focal leakage was detected in the peripapillary and central macular areas of the right and left eyes, respectively. On mid-phase ICGA, extensive hyperfluorescence was observed in both eyes. On SD-OCT, RPE exhibited a “saw-tooth” pattern, and persistent subretinal fluid (SRF) was observed in the peripapillary and central macular areas of the right and left eyes, respectively. A volume scan of the macular area revealed an RPE defect corresponding to focal leakage in the left eye. The central retinal thicknesses were 197 and 359 μm in the right and left eyes, respectively. The subfoveal choroidal thicknesses were 228 and 237 μm in the right and left eyes, respectively. Based on multimodal imaging, the patient was diagnosed with cuticular drusen presenting with CSC. In the patient’s right eye, persistent SRF did not involve the macular area; therefore, treatment was not administered. As persistent SRF involved the fovea in the left eye, photodynamic therapy (PDT) was administered. Two-third dose PDT was administered as previously described by Tanaka et al,^[12]^ and the greatest linear dimension and spot size were found to be 3500 and 4500 μm, respectively, to cover the leakage points.

**Figure 2.**
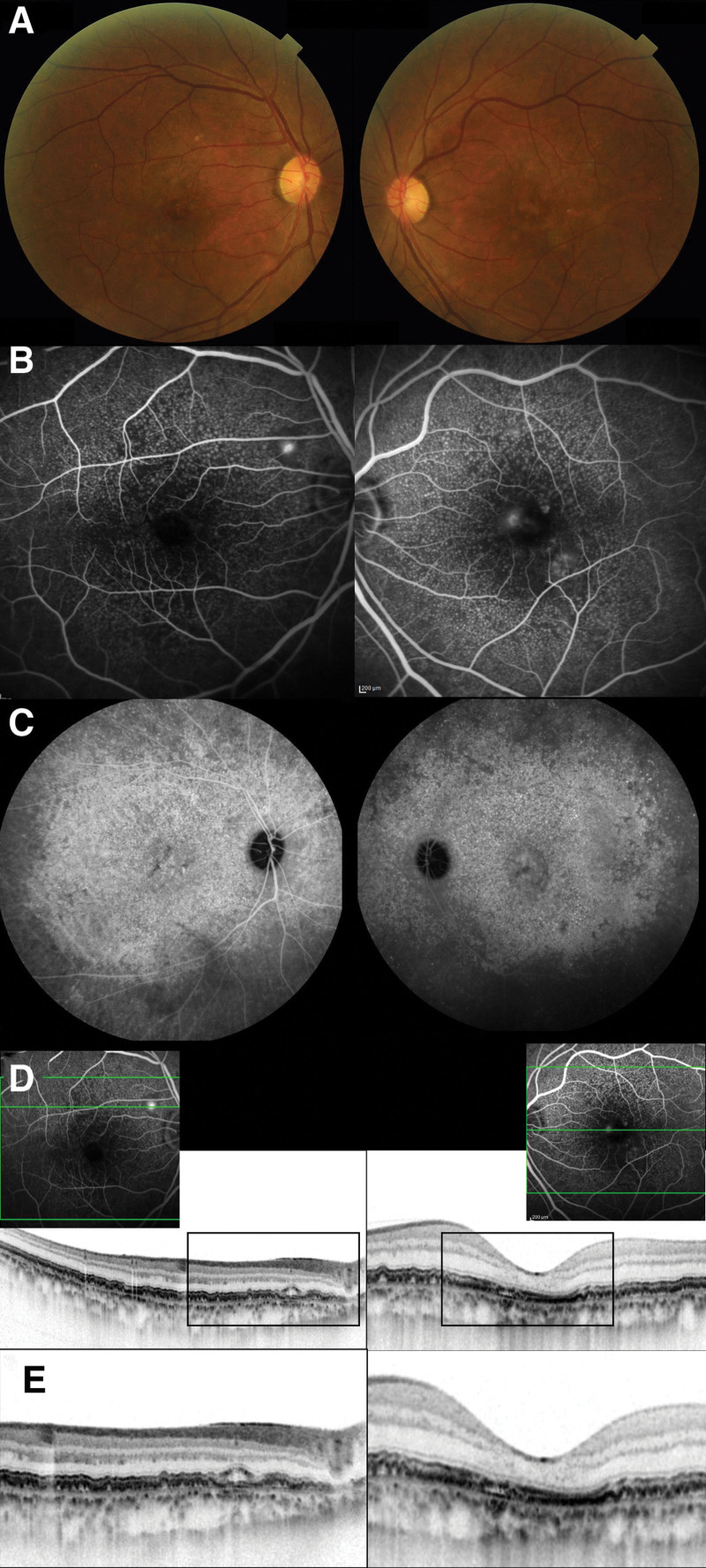
Multimodal imaging 3 months after photodynamic therapy for central serous chorioretinopathy in the left eye. (A) Color fundus photography revealed RPE abnormalities in both eyes, as in the initial presentation. (B) Mid-phase FA revealed leakage from the peripapillary region of the right eye and staining in the left eye. (C) Late-phase wide-field ICGA exhibited hyperfluorescence around the macular areas of both eyes. (D) Horizontal SD-OCT revealed residual subretinal fluid in the right eye and a dry macula in the left eye. (E) Magnified SD-OCT imaging demonstrated that an RPE defect persisted despite the achievement of a dry macula in the left eye. The central retinal thicknesses were 205 and 166 μm in the right and left eyes, respectively. The subfoveal choroidal thicknesses were 239 and 127μm in the right and left eyes, respectively.

At the 1-month visit, the SRF had disappeared in the left eye. At the 3-month visit, we performed a comprehensive examination, as in the initial visit (Fig. 2). On FA, focal leakage was still observed in the right eye, and staining corresponded to leakage points in the left eye. Wide-field late-phase ICGA exhibited hyperfluorescence corresponding to numerous cuticular drusen. SD-OCT revealed that SRF persisted in the peripapillary region; however, the macula was dry in the right eye, and the central macular area completely achieved dryness in the left eye. The BCVA values were 1.2 and 1.0 in the right and left eyes, respectively. In the right eye, the central retinal and subfoveal choroidal thicknesses were 205 and 239 μm, respectively, as were the values at the initial visit. In the left eye, the central retinal and subfoveal choroidal thicknesses decreased to 166 and 127 μm, respectively.

The patient provided the written informed consent for publication.

## 3. Discussion

To the best of our knowledge, this is the first study to report the development of CSC in eyes with cuticular drusen. In this case, PDT was performed for CSC in the left eye because of persistent SRF in the macular region, resulting in a dry macula. Since SRF did not involve the central macula, no treatment was administered to the right eye.

Among several drusen types, cuticular drusen possess unique characteristics in terms of early onset, association with acquired vitelliform lesions, and multimodal imaging. Similar to conventional drusen, cuticular drusen are also associated with advanced AMD, including MNV and GA. Longitudinal studies have demonstrated that the 5-year cumulative incidence rates of MNV and GA are 24 to 28% and 9 to 13%, respectively.^[13,14]^

CSC is a multifactorial disease that involves genetic predisposition and environmental factors. Although the etiology of CSC is not fully understood, choroidal dysfunction is considered the primary cause. CSC is categorized as pachychoroid disease. In addition to focal or diffuse increased choroidal thickness, choroidal vascular hyperpermeability and the presence of pachyvessels are diagnostic criteria for pachychoroid disease. In this case, the subfoveal choroidal thicknesses were 228 and 237 μm in the right and left eyes, respectively. Although late-phase ICGA revealed hyperfluorescence around the macular area, it was not due to choroidal vascular hyperpermeability, but cuticular drusen. The patient did not exhibit pachychoroid characteristics. In the left eye, a large RPE defect was detected in the macular region on SD-OCT, corresponding to leakage on FA. Therefore, in this case, the mechanism underlying CSC development might have differed from that of pachychoroid-associated CSC.

Several therapeutic options have been reported for CSC. Among them, PDT proves effective for the resolution of SRF and improvement of BCVA with decreased choroidal thickness. Three months after two-third dose PDT, BCVA improved from 0.9 to 1.0, with the achievement of dry macula and decreased subfoveal choroidal thickness in the left eye. Half-dose PDT is reportedly effective for the resolution of SRF and improvement of BCVA; however, one-third dose PDT may fail to achieve a dry macula, leading to persistent SRF. Recently, Tanaka et al demonstrated that two-third dose PDT is effective for the resolution of exudation in eyes with pachychoroid diseases.^[12]^ Therefore, we administered two-third dose verteporfin when performing PDT for eyes with CSC.

The development of MNV is a serious ocular adverse event after PDT in eyes with CSC, and several cases have been documented.^[15–17]^ Recent reports have demonstrated that older age and the presence of baseline shallow irregular pigment epithelial detachment are risk factors for MNV development in eyes with CSC after PDT.^[16,17]^ Although no baseline shallow irregular pigment epithelial detachments were detected in this case, the patient was considered to be at high risk of AMD development. Therefore, a longer follow-up period and careful examination are required in this case.

## Author contributions

**Conceptualization:** Yoichi Sakurada.

**Data curation:** Shuhei Hosoda, Yoichi Sakurada, Taiyo Shijo.

**Supervision:** Kenji Kashiwagi.

**Writing—original draft:** Shuhei Hosoda.

**Writing—review and editing:** Yoichi Sakurada, Taiyo Shijo, Kenji Kashiwagi.
